# Parental kinship influences global methylation and epigenetic age estimation in *Peromyscus*

**DOI:** 10.1093/genetics/iyaf281

**Published:** 2025-12-29

**Authors:** Kim-Tuyen Huynh-Dam, Celia Jaeger, Ioulia Chatzistamou, Steve Horvath, Hippokratis Kiaris

**Affiliations:** Department of Drug Discovery and Biomedical Sciences, College of Pharmacy, University of South Carolina, Columbia, SC 29208, United States; Peromyscus Genetic Stock Center, University of South Carolina, Columbia, SC 29208, United States; Department of Pathology, Microbiology and Immunology, School of Medicine, University of South Carolina, Columbia, SC 29208, United States; Department of Human Genetics, David Geffen School of Medicine, University of California Los Angeles, Los Angeles, CA 90095, United States; Altos Labs, Institute of Science, Cambridge CB21 6GP, UK; Department of Drug Discovery and Biomedical Sciences, College of Pharmacy, University of South Carolina, Columbia, SC 29208, United States; Peromyscus Genetic Stock Center, University of South Carolina, Columbia, SC 29208, United States

**Keywords:** epigenetic, ageing, kinship, inbreeding, *Peromyscus*

## Abstract

Kinship relationships between parents affect offspring fitness. Beyond its effects in heterozygosity or its impact in deleterious alleles that can be reduced to homozygosity and decrease the individuals' fitness, the consequences of parental relatedness in the offspring remain understudied. By leveraging the availability of detailed pedigrees of captive *Peromyscus*, we explored how parental relatedness impacts the methylome and the epigenetic age estimation of the offspring. Global CpG methylation analysis showed that parental relatedness positively impacts lifespan expectancy and reduces epigenetic ageing, contributing about 13% of variancein epigenetic age estimation. Global hypermethylation due to relatedness was considerably higher than hypomethylation, was more pronounced in the male offspring, and mainly affected chromosomal loci associated with development. A relatedness-associated methylation signature was described that predicts parental relatedness with high accuracy, providing the proof of concept that kinship relationships can be inferred by epigenetic analyses. These findings identify parental relatedness as a modifier of epigenetic ageing and global methylation, suggesting that kinship relations should be considered when epigenetic, and potentially transcriptomic data are interpreted in the context of ageing and of other pathophysiological processes.

## Teaser

Kinship relationships between parents affect offspring fitness. By leveraging the availability of detailed pedigrees of captive *Peromyscus* Huynh-Dam et al. explored how parental relatedness impacts the methylome and the epigenetic age estimation of the offspring. Their analyses identify parental relatedness as a modifier of epigenetic aging and global methylation, suggesting that kinship relations should be considered when epigenetic, and potentially transcriptomic data are interpreted in the context of aging and of other pathophysiological processes.

## Introduction

Inbreeding is common in animal and small human communities that are isolated due to cultural and geographical reasons. Inbreeding may lead to inbreeding depression by mechanisms involving reduction of deleterious alleles to homozygosity or loss of the heterozygote advantage (Charlesworth and Willis 2009). The impact of heterozygosity has been studied extensively in wild populations and has been linked intrinsically to the conservation of threatened and endangered species, being recognized as an important determinant of their fitness (DeWoody et al. 2021). Overall heterozygosity may also be of particular significance in human populations considering that several small communities around the world, due to their geographical and cultural isolation, are characterized by increased longevity and favorable health outcomes despite that inbreeding could have reduced the individuals' fitness (Mitchell et al. 2001; Panagiotakos et al. 2011; Buettner and Skemp 2016; Partridge et al. 2018; Schmidt 2019; Rodríguez-Girondo et al. 2021).

To explore this question, we used *Peromyscus* (deer mice) as a model. Different deer mouse stocks are maintained for several decades as closed colonies at the Peromyscus Genetic Stock Center (PGSC) (Havighorst et al. 2017). The animals are maintained as genetically diversified stocks through random breeding at regulated animal facility conditions, without selecting the animals that are chosen to serve as breeders (Lucius et al. 2021; Huynh-Dam et al. 2025a). Breeding records are available for all mice that are born at the PGSC which allows calculation of the animals' parental relatedness and estimation of their kinship based on pedigree analyses (Havighorst et al. 2017; Huynh-Dam et al. 2025b). These animals exhibit sensitivity to seasonal changes in breeding and methylation suggesting that they retain their wild type characteristics in captivity (Huynh-Dam et al. 2025a). By combining pedigree analyses for estimation of parental kinship with comprehensive analyses of the epigenome we show that parental relatedness alters the methylome and reduces the animals' epigenetic age mitigating the effects of inbreeding depression.

## Methods

### Breeding

Breeding of deer mice is performed in accordance with Institutional guidelines IACUC approval # 2644-101795-042823. Animals are kept in a 16 h light/8 h dark cycle throughout the year. Animals were maintained under standard animal facility conditions, by establishing randomly, on a rotational basis, breeding pairs with adult animals older than 2 months old, that are maintained as breeders for at least 1.5 to 2 years, until their productivity is reduced and are substituted by others. This happens throughout the year, by a manner that is not purposely discriminatory.

### Relatedness score and differential gene/methylation studies

The relatedness score was analyzed using kinship2 package (Sinnwell et al. 2014). The database of Peromyscus colonies was maintained in Microsoft Access and exported to excel files for bioinformatic analysis (available upon request). The analysis involved tail DNAs from 96 virgin *Peromyscus maniculatus* (47 females and 49 males) of ages spanning from 1.3 to 33 months and was conducted by the Clock Foundation (Torrance, CA) as described earlier (Horvath et al. 2022; Li et al. 2024). Briefly, tail DNA was isolated, subjected to bisulfite conversion, and analyzed for DNA methylation by using the HorvathMammalMethyl40 array that assays methylationfor up to 37,000 conserved CpGs dispersed across mammalian genomes (Horvath et al. 2022; Li et al, 2024) Epigenetic age and calculations of predicted lifespan were adjusted for average chronological age using the formula CorrectedAge = DNAmAge X (AvAge/AvDNAmAge) whereas AvAge and AvDNAmAge are the average chronological and DNAmAge of the dataset. Validation of epigenetic age estimation was performed using a second, independent dataset with animals that were born 4 to 5 years before the animals of the first group with no common animals between the two. The raw data of DNA methylation of this second dataset was retrieved from previous publication (Horvath et al. 2022) and included only the tail samples from virgin animals (*n* = 86) (Horvath et al. 2022).

Raw DNA methylation data were processed using the SeSAMe (SEnsible Step-wise Analysis of Methylation data) pipeline (Zhou et al. 2018). This method applies background correction, dye bias correction, and probe masking to generate high-quality normalized beta values. The resulting SeSAMe-normalized dataset was used for the analysis of differential methylated CpGs associated with parental relatedness score using Likelihood Ratio Test to compare nested linear models—a full model that incorporates relatedness score and a reduced model that excludes it—while also controlling for age and sex as confounding factors (Crowgey et al. 2018; Karlsson et al. 2018).

- Reduced model:
  $$\mathrm{ProMe}t_{ij}=\beta_{0}+\beta_{1}\text{·}\mathrm{Ag}e_{i}+\beta_{2}\text{·}\mathrm{Se}x_{i}+\varepsilon_{ij}$$
- Full model:
  $$\mathrm{ProMe}t_{ij}=\beta_{0}+\beta_{1}\text{·}\mathrm{Ag}e_{i}+\beta_{2}\text{·}\mathrm{Se}x_{i}+\beta_{3}\text{·}\mathrm{Relatednes}s_{i}+\varepsilon_{ij}$$

### Feature selection and predictive modeling

To develop a robust methylation signature of parental relatedness, we employed a multistage modeling approach. First, we filtered the dataset to retain only those CpGs identified as significant (FDR < 0.05) from the differential methylation analysis described above. Second, to reduce dimensionality and eliminate redundancy among these candidates, we performed LASSO regression (*α* = 1) using 10-fold cross-validation (Tibshirani 1996). CpGs with nonzero coefficients were retained for the final modeling stage. The dataset was then split into 80% training and 20% testing. Using the features selected by LASSO, we trained a final Elastic Net regression model (*α* = 0.5). The input features for this model consisted of the continuous methylation Beta values for the selected CpGs. Elastic Net was chosen to balance the sparsity of LASSO with the Ridge penalty, thereby better handling collinearity among correlated CpG sites (Zou and Hastie 2005 ). The optimal λ was determined via cross-validation on the training set. Model performance was evaluated using root mean squared error (RMSE) and *R*² on the test set. A scatter plot of observed vs. predicted relatedness was generated to visually assess model accuracy. A linear regression line was fitted to illustrate agreement between predictions and actual values.

### Epigenetic age acceleration

Epigenetic age acceleration (EAA) was defined as residuals from linear regression of DNAmAge on chronological age and sex (Horvath 2013).

### Regression of relatedness on epigenetic age acceleration

The relationship between relatedness and EAA was assessed by ordinary least squares (OLS) regression, with robustness checks using heteroskedasticity-consistent SEs (HC3) (Long and Ervin 2000) and robust regression with M-estimation (Huber 1972; Rousseeuw and Leroy 1987). Added-variable (partial regression) plots visualized the adjusted association. Leave-one-out (jackknife) analyses sequentially excluded each sample to test robustness.

### Epigenetic entropy

Global methylome entropy was calculated using a kernel density estimator (KDE)-based Shannon entropy (Jenkinson et al. 2017). Associations with age, relatedness, and EAA were tested by linear models, with supplementary residual analyses performed to account for confounders.

### Principal component analysis

Principal component analysis (PCA) was performed on normalized β-values to capture structured methylome variation. The top components were examined for associations with relatedness, sex, and residual DNAmAge.

### Mediation analysis

Causal mediation analysis tested whether PCs mediated the effect of relatedness on EAA, using the *mediation* package (Imai et al. 2010; Tingley et al. 2014). Models estimated the average causal mediation effect (ACME, indirect), average direct effect (ADE), total effect, and proportion mediated. Significance was assessed by nonparametric bootstrapping with 10,000 simulations to ensure precise estimation of confidence intervals. Sensitivity analyses evaluated robustness under violations of the sequential ignorability assumption.

### Sex-stratified mediation

To explore sex-specific effects, mediation models were run separately for males and females. Subgroup results are reported in Supplementary Tables.

### Distribution of CpGs in different chromosomes

To determine if relatedness-dependent CpGs were nonrandomly distributed across the genome, we performed an enrichment analysis for each chromosome. For every chromosome (1 to 23 and X), we constructed a 2 × 2 contingency table comparing the number of significant relatedness-associated CpGs (FDR < 0.1) and nonsignificant CpGs located on that chromosome vs. the corresponding counts in the rest of the genome. Statistical significance of enrichment or depletion was assessed using Fisher's Exact Test to calculate exact probabilities of deviation from the background distribution. To control for the false discovery rate associated with multiple testing across 24 chromosomes, *P*-values were adjusted using the Benjamini–Hochberg (BH) procedure. Chromosomes with an adjusted *P*-value (*q*-adj) < 0.05 were considered significantly enriched or depleted.

### Sub-chromosomal enrichment analysis and gene mapping

To investigate the spatial distribution of differentially methylated CpGs at a sub-chromosomal scale, we performed a sliding window enrichment analysis. The genome was divided into 2-Mb windows based on the coordinate annotations of the MammalMethylChip40 array. Within each window, we calculated the proportion of relatedness-associated CpGs (identified as FDR < 0.05 in the differential methylation analysis) relative to the total number of assayed CpGs in that window. Enrichment significance was assessed using a hypergeometric test, comparing the local density of significant CpGs to the genome-wide background rate. *P*-values were adjusted for multiple comparisons using the BH (FDR) method, and windows with an FDR < 0.05 were considered significantly enriched. To characterize the biological relevance of these clusters, genes located within the significant windows were mapped using the array annotation. Specifically, genes directly associated with the differentially methylated CpGs driving the window's enrichment were identified as candidate drivers of the regional methylation changes (e.g. Zic1, Gsx2).

### Software

Analyses were conducted in R (R Core Team 2025) using RStudio (RStudio Team 2025). Figures were produced with *ggplot2* (Wickham 2016) and Prism GraphPad (GraphPad Software, San Diego, CA).

### Gene ontology analysis

Gene Ontology (GO) analysis was performed using ShinyGO 0.77 (http://bioinformatics.sdstate.edu/go/). The top enrichment was selected by FDR, sorted by fold enrichment.

## Results

### Parental relatedness delays epigenetic ageing

**Fig. 1.**
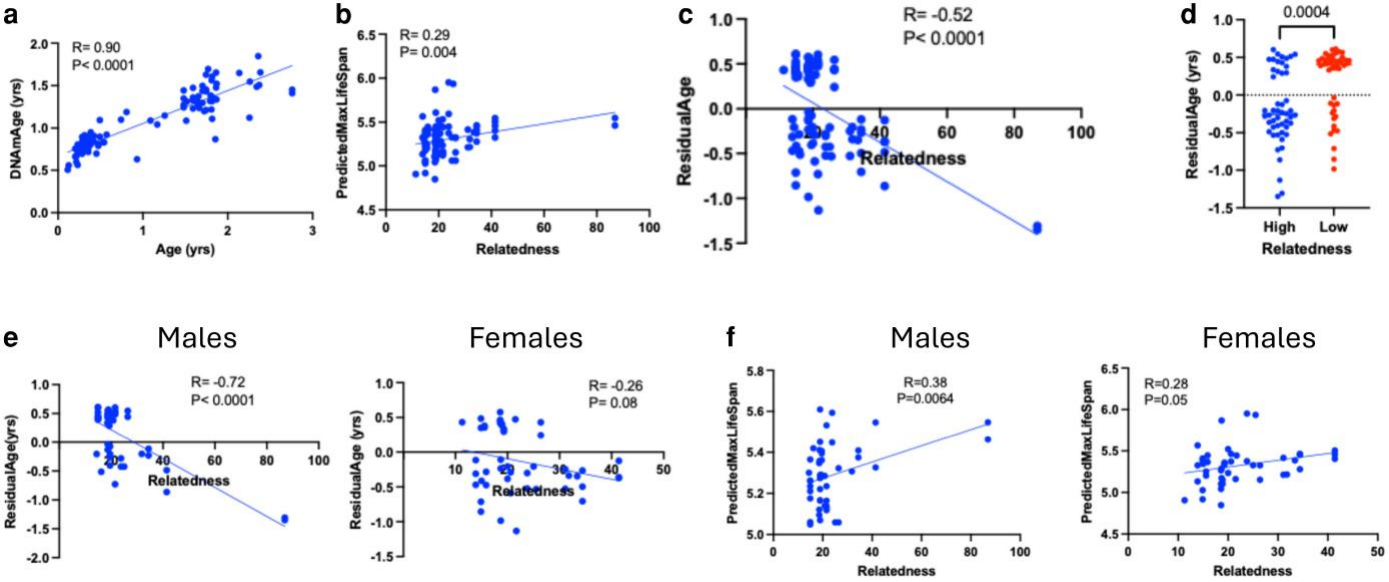
Methylation clock for *P. maniculatus* and predicted lifespan. a) Validation of the epigenetic clock for *P. maniculatus* (BW stock). The *x*-axis shows the animals' biological age while the *y*-axis shows the predicted epigenetic age (DNAmAge) of the animals. b) Scatter plot showing the correlation between predicted lifespan (*y*-axis) and relatedness (*x*-axis). c) Correlation between age residual and relatedness in *P. maniculatus*. d) Residual age in animals having equal or above (high) (*n* = 50) or below (low) (*n* = 46) median relatedness are shown. *P*-value (Student's t-test) is indicated. e) Correlation between age residual (*y*-axis) and parental relatedness (*x*-axis) in male or female *P. maniculatus*. f) Scatter plot showing the correlation between predicted lifespan (*y*-axis) and relatedness (*x*-axis) in males (left) and females (right) *P. maniculatus*. *P* and R values (Pearson's correlation) are shown in the scatter plots. Epigenetic age and calculations of predicted lifespan were adjusted for average chronological age (see Methods for more details).

To evaluate the impact of parental relatedness in lifespan expectancy, we analyzed epigenetic age estimators that accurately predict chronological age (Haghani et al. 2023; Lu et al. 2023). To that end we performed a new whole genome methylation analysis in a cohort of 96 *P. maniculatus* (47 females and 49 males) of ages spanning from 1.3 to 33 months and estimated their epigenetic age as described (Horvath et al. 2022). The validation of the methylation clock from this dataset is shown in Fig. 1aindicating highly significant correlation between chronological and epigenetic age. By using this dataset, we discovered that parental relatedness positively correlated with the animals' predicted maximal lifespan (*R* = 0.29, *P* = 0.004) implying that parental kinship influences the epigenetic age estimation in the offspring (Fig. 1b).

The residual age, that reflects the difference between epigenetic age and chronological age, was significantly impacted by parental relatedness. In scatter plots depicting residual age and parental relatedness, an inverse correlation was unveiled suggesting that ageing was decelerated in the inbred animals (Fig. 1, c and d). Furthermore, the residual age of the offspring of parents exceeding the median relatedness of the group was significantly lower than the offspring of parents that had relatedness score higher than the group's median (Fig. 1d). Independent assessment of the cohort of males and females showed that in the former, the association remained highly significant (*R* = −0.72, *P* < 0.0001) but not in the latter (*R* = −0.26, *P* = 0.08) suggesting that male *P. maniculatus* offspring are more sensitive than the female offspring, to the modulation of their epigenetic age by parental relatedness (Fig. 1e). Consistently with the inverse effect of relatedness in residual age, a positive effect of relatedness in predicted maximum lifespan in male (*R* = 0.38; *P* = 0.0064) and female (*R* = 0.28; *P* = 0.053) *P. maniculatus* was estimated (Fig. 1f). Removal of the two outliers exhibiting very high relatedness and reanalysis of the data resulted in similar results (Supplementary Fig. 1).

To confirm these findings in an independent dataset, we revisited previously reported epigenetic data by re-analyzing the data after retrieving their pedigree records and calculating their parental relatedness (Horvath et al. 2022). The group included 47 females and 39 males from different *Peromyscus* stocks: The polygamous *P. leucopus* (LL) and two polygamous stocks of *P. maniculatus* (BW and SM2)], and the monogamous *P. californicus* (IS), *P. eremicus* (EP), and *P. polionotus* (PO) deer mouse species. For this analysis, only virgins were considered to exclude potential confounding effects of reproduction in epigenetic age calculations. The group involved animals from all stocks available at the PGSC, as well as 6 (3 males and 3 females) F1 hybrids between *P. maniculatus* (BW), and *P. polionotus* (PO) (Horvath et al. 2022). These animals were born 4–5 years before the animals of the first group with no common animals between the two. Analysis of this earlier reported dataset confirmed that increased parental relatedness also predicts reduced epigenetic age (Supplementary Fig. 2). Analysis of males and females showed similar trends but remained nonsignificant (Supplementary Fig. 2). The lower significance unveiled in this dataset compared to the one involving *P. maniculatus* (BW stock) only, can be either due to the samples' stock diversity (6 stocks and 6 individuals F1 PO X BW hybrids) or because this behavior is unique for *P. maniculatus*.

### Parental kinship does not predict relatedness between animals

**Fig. 2.**
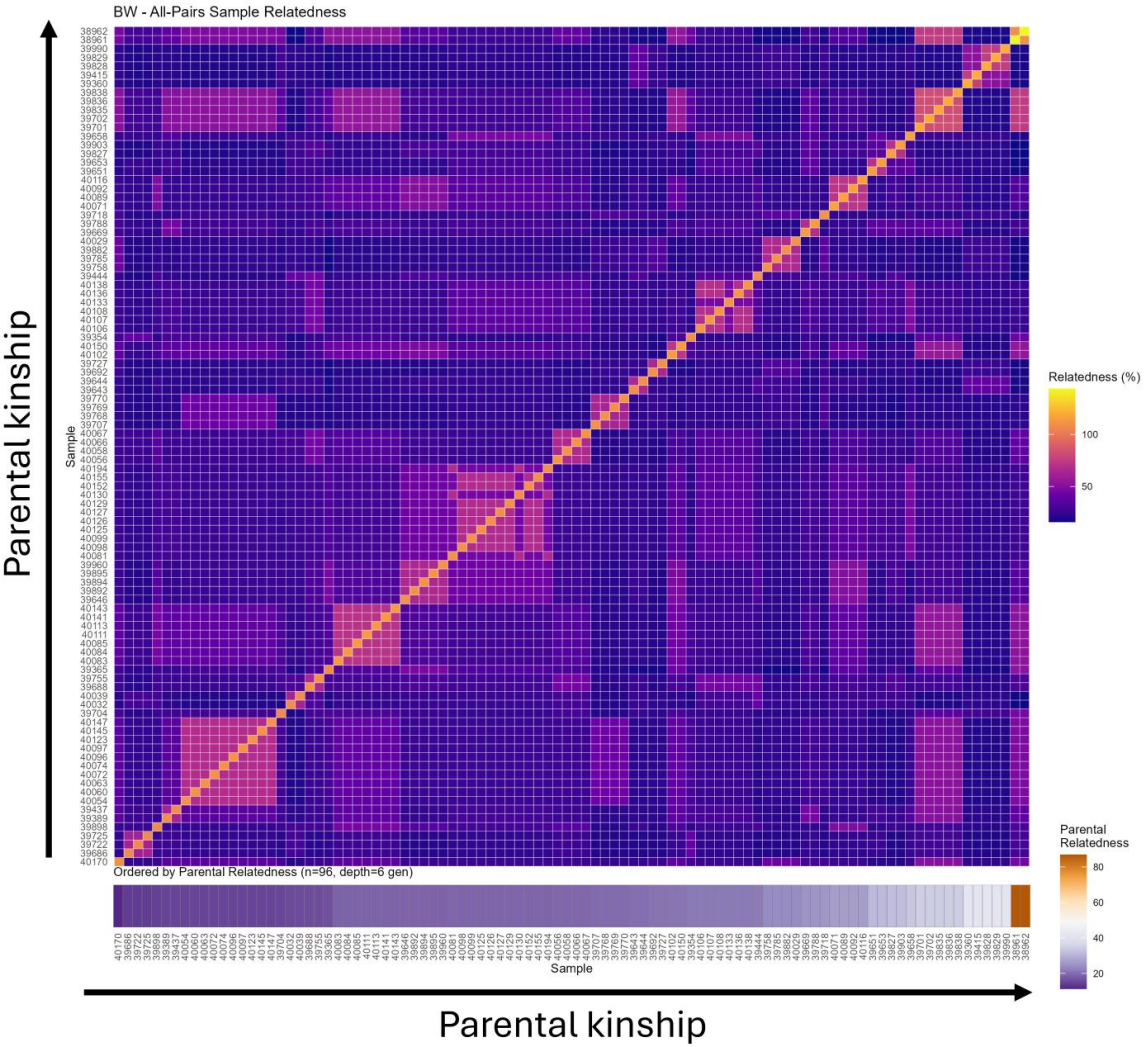
Parental kinship and offspring relatedness. Heatmap showing the relatedness of each animal in the cohort, arranged by increased parental relatedness.

In view of the fact that the animals included in this analysis correspond to a closed inbred colony, the effects of parental relatedness may be confounded by genetic variation. From this standpoint, the specific impact of parental relatedness may not be due to relatedness *per se* but rather due to shared alleles between related parents that are also related to other related parents that passed down these alleles to their offspring. If this was the case, we would expect from related parents to produce offspring that are also more related to each other (since they are all offspring of related parents). To address this possibility, we have generated a matrix showing the relatedness of each animal in the cohort, arranged by increased parental relatedness. As shown in Fig. 2, no noteworthy clustering of samples emerged, and increases in parental relatedness did not result in increases in the relatedness between the animals, which in turn suggests that the effects of parental relatedness are not due to genetic variation.

### Added-variable regression establishes robust negative association between relatedness and epigenetic age acceleration

**Fig. 3.**
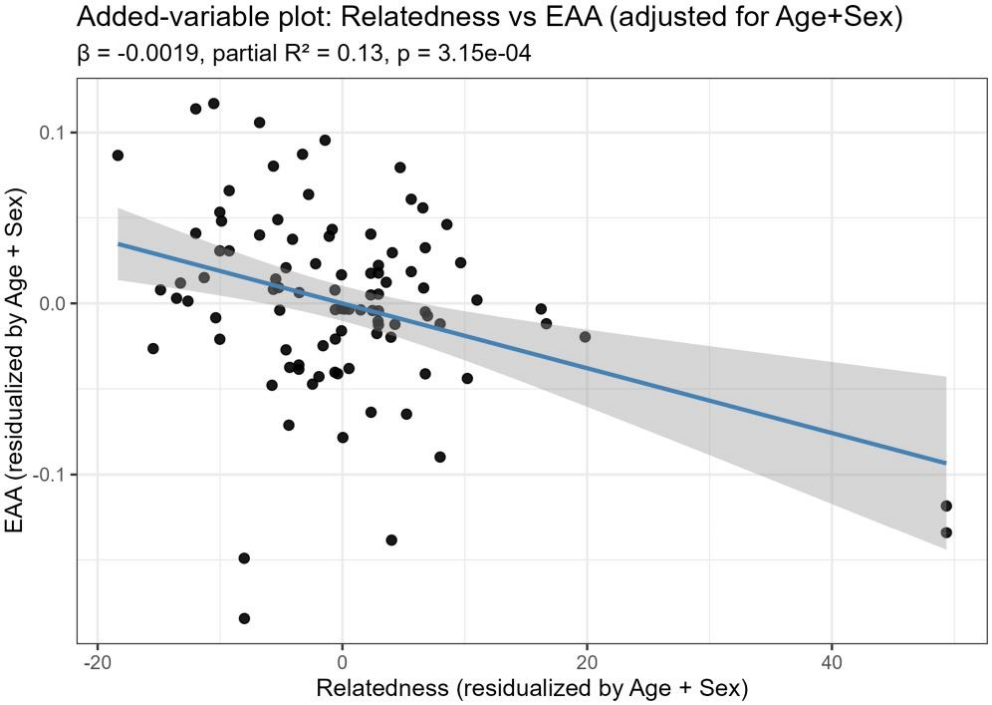
Added-variable (partial regression) plot showing the association between relatedness and EAA, adjusted for age and sex.

To rule out that the impact of relatedness in epigenetic age estimation was confounded by the animals' sex and age, we calculated EAA, adjusting for age and sex. Regression analysis showed significant negative association (*β* = −0.00145, *P* = 0.0018), which remained stable when using heteroskedasticity-consistent standard errors (HC3; *β* = −0.00145, *P* = 0.0012) and robust regression with M-estimation (*β* = −0.00151, *P* < 0.001), confirming robustness to heteroskedasticity and outliers (Huber 1972; Rousseeuw and Leroy 1987; Long and Ervin 2000). As illustrated in Fig. 3, relatedness and EAA were inversely correlated, with relatedness explaining ∼13% of residual variance after accounting for age and sex. Leave-one-out analyses confirmed that this association was not driven by single individuals (median β ≈ −0.00189, IQR [−0.00206; −0.00162], Supplementary Fig. 3, Supplementary Table 1), confirming the robust negative association between relatedness and EAA.

### Epigenetic entropy reflects age-related methylome disorder

Entropy, a measure of global methylome disorganization, increased with chronological age (R² ≈ 0.158, *P* < 0.0001; Supplementary Fig. 4a), consistent with previous reports of entropy-driven epigenetic drift in mammals (Jenkinson et al. 2017; Lowe et al. 2018). In line with this, EAA and entropy were positively correlated, independently of relatedness, age and sex (partial R² ≈ 0.376, *P* < 0.0001; Supplementary Fig. 4b). Residual analyses confirmed the lack of significant association between entropy and relatedness after adjusting for age and sex (partial R² ≈ 0.011, *P* = 0.308; Supplementary Fig. 4c). Thus, entropy captures age-driven stochastic methylome variation rather than genetic similarity.

### Principal components capture structured methylome variation linked to relatedness and EAA

**Fig. 4.**
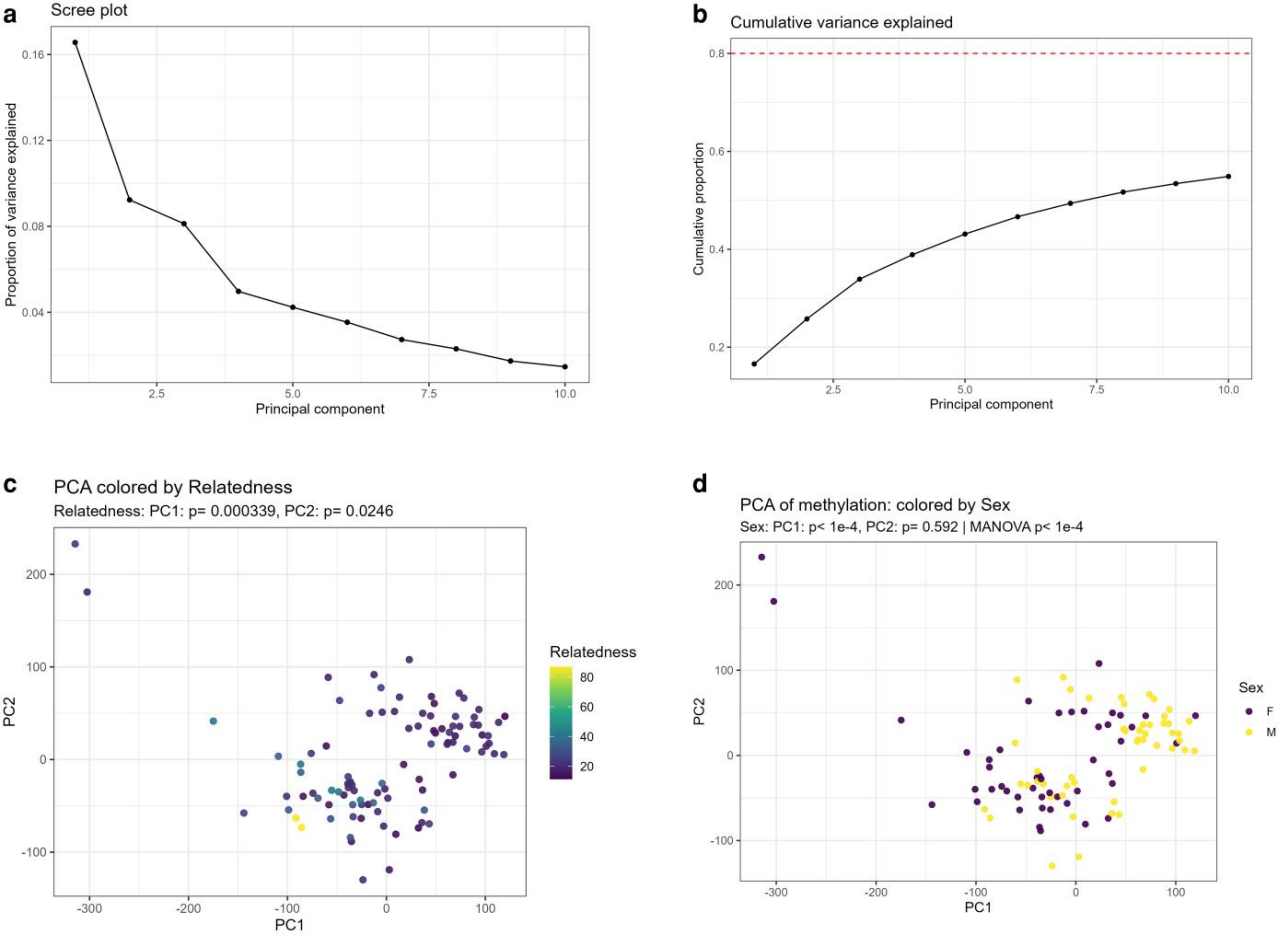
Principal components capture structured methylome variation. a) Scree plot of variance explained by the first 10 principal components (PCs). b) Cumulative variance explained (dashed line marks the 80% reference); the first three PCs account for ∼35% of total variance. c) PC1 vs. PC2 colored by Relatedness. Relatedness is associated with PC1 (*P* = 3.39 × 10⁻⁴) and modestly with PC2 (*P* = 0.0246). d) PC1 vs. PC2 colored by Sex. Sex is associated with PC1 (*P* < 10⁻⁴) but not PC2 (*P* = 0.592); jointly, PC1 + PC2 differ by Sex (MANOVA, Pillai *P* < 10⁻⁴). Points are individuals; axes are standardized PC scores. Per-PC *P*-values come from linear models (PCk ∼ predictor); the joint test uses MANOVA on cbind(PC1, PC2) ∼ predictor. Color scales are continuous for Relatedness and discrete for Sex.

**Fig. 5.**
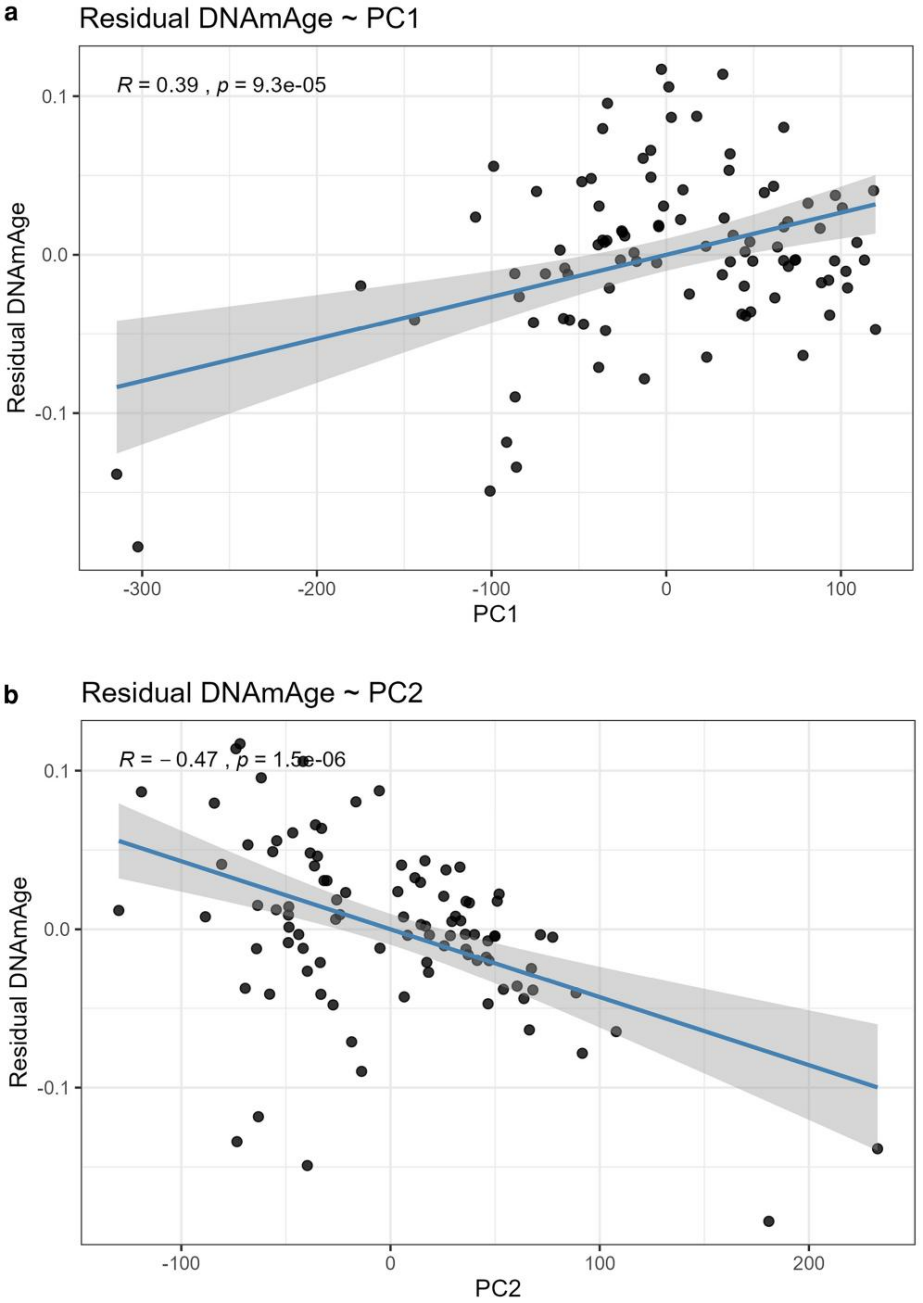
Residual DNAmAge (EAA) relates to leading PCs. a) Residual DNAmAge (EAA) vs. PC1 with least-squares fit and 95% CI. Pearson *R* = 0.39, *P* = 9.3 × 10⁻⁵. b) Residual DNAmAge (EAA) vs. PC2 with least-squares fit and 95% CI. Pearson *R* = −0.47, *P* = 1.5 × 10⁻⁶.

While entropy captures stochastic methylome disorganization, principal components (PCs) summarize structured, nonrandom axes of methylome variation. Unlike entropy, which reflects noise accumulation with age, PCs extract latent factors that represent coordinated CpG covariation across the genome (Jenkinson et al. 2017). This makes them ideal mediators because they can capture methylome structure influenced by parental relatedness and, in turn, propagate effects onto epigenetic ageing. Indeed, PC approaches have been widely used to summarize DNA methylation structure and correct for confounding or reveal biological signatures in Epigenome-Wide Association Studies (EWAS) (Leek and Storey 2007; Jaffe and Irizarry 2014; Rahmani et al. 2019). Thus, some factors, such as sex and age, which are mostly explaining PC1 and/or PC2, are the biologically meaningful mediators. PC analysis (PCA) identified major axes of methylome structure, with the first three PCs explaining ∼35% of variance (Fig. 4, a and b). Relatedness is associated mainly with PC1 (*P* = 3.39 × 10⁻⁴) and to a lesser extent with PC2 (*P* = 0.0246), whereas Sex is only associated with PC1 (*P* < 10⁻⁴) but not PC2 (*P* = 0.592); jointly, PC1 + PC2 differ by Sex (MANOVA *P* < 10⁻⁴; Fig. 4, c and d). Residual DNA methylation Age (DNAmAge) was strongly associated with both PC1 and PC2 (Fig. 5, a and b), indicating that structured methylome variation influences clock-based ageing estimates.

### The effect of relatedness on EAA is largely mediated by structured methylome variation

**Fig. 6.**
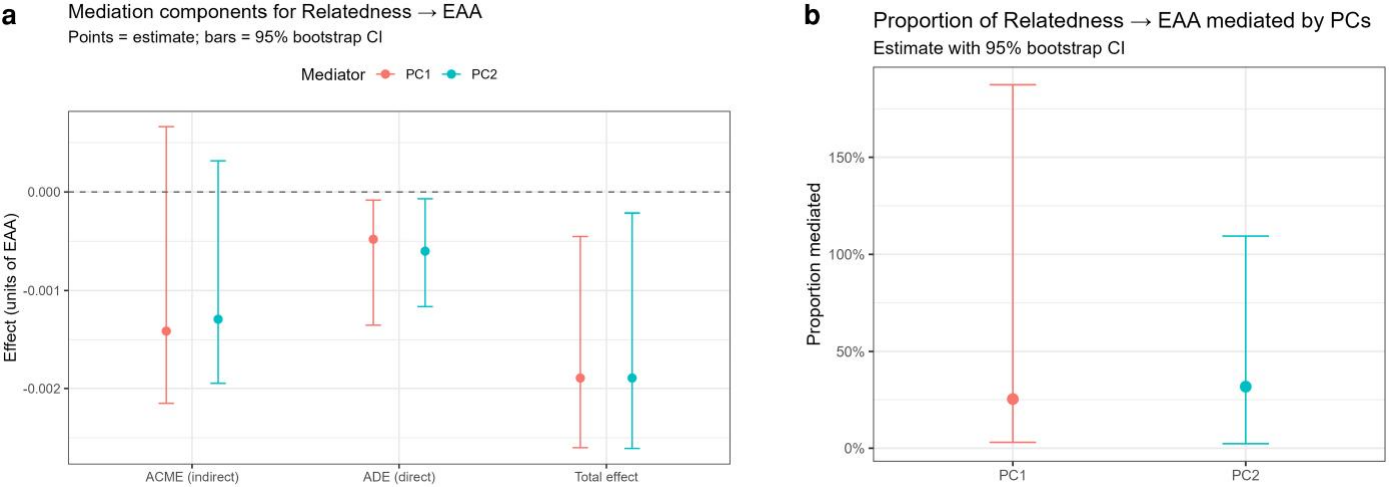
Mediation reveals indirect pathways from relatedness to EAA via methylome PCs. a) Mediation components. Decomposition of the association Relatedness → EAA into the average causal mediation effect (ACME; indirect), the average direct effect (ADE; direct), and the Total effect. Points show estimates; bars denote 95% nonparametric bootstrap CIs (10,000 draws); horizontal dashed line = 0. PC1 (red) and PC2 (blue) each exhibit a significant ACME (indirect effect < 0), whereas the ADE is weaker and close to zero. b) Proportion mediated. Contribution of each PC to the total effect, computed as ACME/Total from the same mediation run and displayed with 95% bootstrap CIs. PC1 accounts for ∼25% of the total effect and PC2 for ∼32%.

Causal mediation analysis (Imai et al. 2010; Tingley et al. 2014) demonstrated that the association between relatedness and EAA is partially explained by structured methylome variation. Figure 6a shows the decomposition of the effect of relatedness on EAA into direct and indirect pathways. For both PC1 (red) and PC2 (blue), the ACME was significantly different from zero, while the ADE was weaker.

Quantification of the contribution of each PC to the total effect of relatedness on EAA showed that PC1 accounted for approximately 25% of the total effect, while PC2 explained about 32% (Fig. 6b). The robustness of the effects of PC1 and PC2 was further demonstrated by bootstrap distribution (*n* = 10,000) of the ACME that indicated distribution centered below 0, with 95% confidence (Supplementary Figs. 5, a and b). The robustness of these effects was further demonstrated by sensitivity analyses (Imai et al. 2010) which showed that the estimated ACME for both PC1 and PC2 remained consistently negative across a wide range of hypothetical correlations (ρ) between mediator and outcome error terms (Supplementary Fig. 6). Together, these analyses support the notion that relatedness does not act on EAA solely through direct mechanisms. Instead, a substantial proportion of its effect is transmitted indirectly through global methylome variation summarized by PCs. By capturing structured patterns in the methylome, PC1 and PC2 serve as mediators that channel genetic similarity into biological ageing processes.

To better understand the biological basis of these mediating PCs, we examined the top CpG contributors to PC1 and PC2 using functional enrichment analysis (Supplementary Table 2). CpGs driving PC1 were predominantly enriched for developmental and morphogenetic pathways, including neurogenesis, organ morphogenesis, and DNA-binding transcription factor activity (Supplementary Fig. 7a). This pattern suggests that PC1 represents a global developmental methylome program that broadly influences epigenetic ageing. By contrast, CpGs contributing to PC2 were enriched for cis-regulatory and transcriptional processes, such as RNA polymerase II–dependent transcription, RNA biosynthesis, and cis-regulatory DNA binding (Supplementary Fig. 7b). Notably, PC2 also overlapped with a subset of relatedness-associated CpGs (see below for CpGs associated with relatedness), highlighting its role as a mechanistic link between parental kinship and transcriptional regulation that accelerates epigenetic ageing (Supplementary Table 2). Together, these findings indicate that while both PC1 and PC2 mediate the association between relatedness and EAA, they do so through distinct biological routes: PC1 via broad developmental methylome structure and PC2 via transcriptional regulatory pathways sensitive to relatedness.

### Sex-stratified mediation analyses revealed distinct patterns

In females (*n* = 47), PC1 showed an indirect-only effect (ACME = −0.00177, 95% CI [−0.00323, −0.00045], *P* = 0.01), while the direct (ADE = 0.00079, *P* = 0.404) and total effects based on the same model (total = −0.00098, *P* = 0.261) were not significant; PC2 showed no significant mediation (ACME *P* = 0.263). In males (*n* = 49), PC1 exhibited a small direct effect (ADE = −0.00243, 95% CI [−0.00315, −0.00024], *P* = 0.0352), with nonsignificant mediation (ACME *P* = 0.442); by contrast, PC2 mediated the association (ACME = −0.00063, 95% CI [−0.00143, −0.00011], *P* = 0.0328) (Supplementary Fig. 8, Supplementary Table 3). BH q-values (by sex) appear in figure labels. Thus, sex-specific pathways are involved in the mediation of the effects of genetic relatedness in epigenetic ageing, with PC1 being more prominent in females and PC2 in males.

### Relatedness-dependent methylation targets specific CpGs for hypermethylation and impacts developmental processes

**Fig. 7.**
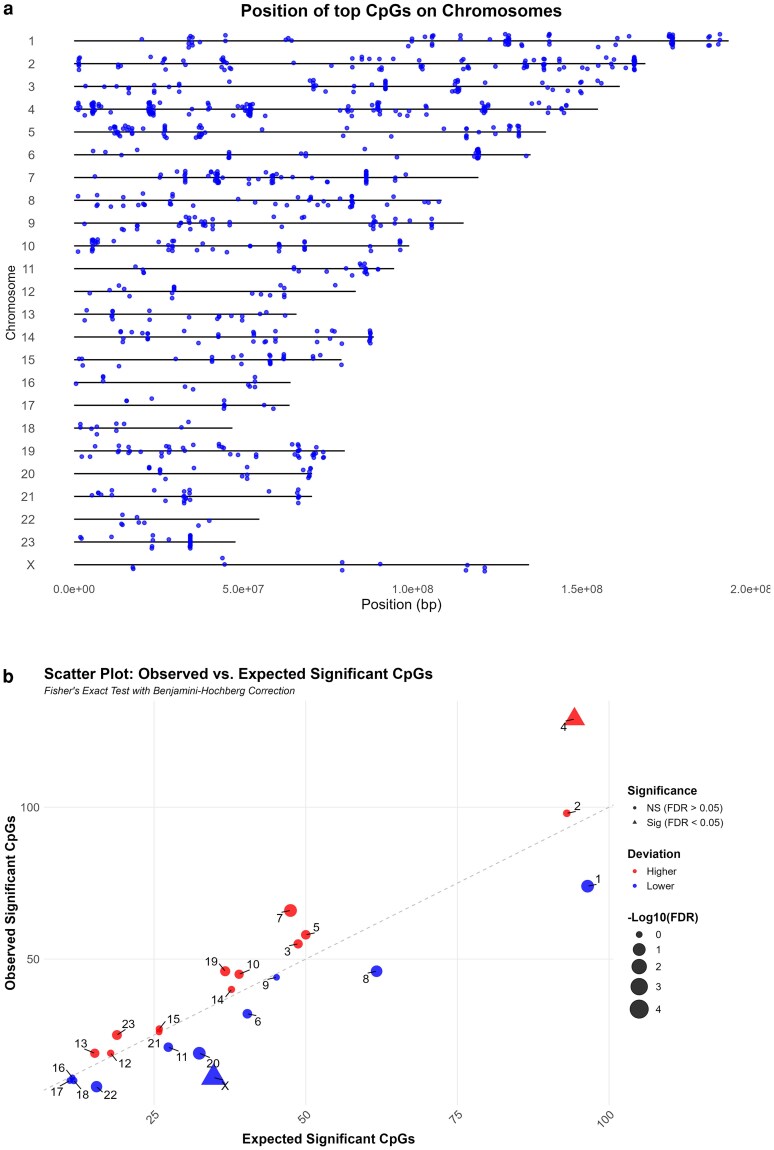
Differentially methylated CpGs according to relatedness. a) Localization of differentially methylated CpGs (FDR < 0.1) in the different *Peromyscus* chromosomes. b) Scatter plot showing the numbers of differentially methylated CpGs in different chromosomes. Blue color indicates under-representation while red color indicates over-representation in the corresponding chromosomes. Significant deviation (chi-square test, *P* < 0.05) is indicated by triangles.

**Fig. 8.**
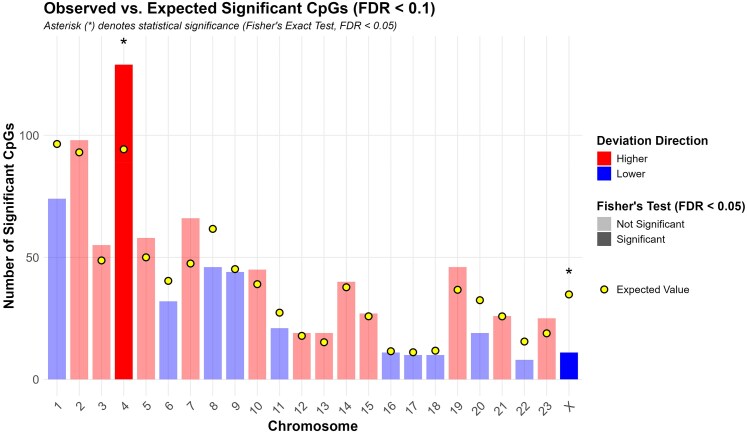
Expected vs. observed CpGs. Bar plot showing the numbers of differentially methylated CpGs in different chromosomes. Blue color indicates under-representation while red color indicates over-representation in the corresponding chromosomes. Yellow circles show number of expected CpGs.

Our findings demonstrate that parental relatedness influences the epigenetic age of *Peromyscus* by driving differential methylation of specific CpGs. This implies that specific CpGs are more sensitive to the effects of parental relatedness. Thus, we sought to identify the CpGs that exhibit relatedness-dependent methylation and evaluated the predicted biological functions of the corresponding genes. By using *P* < 0.05 and FDR < 0.1, we identified 986 CpGs in *P. maniculatus* that exhibited parental relatedness-dependent methylation. The corresponding CpG IDs, adjacent genes, chromosomal location and presence in CpG islands, *P* and FDR values are shown in Supplementary Table 4. Out of these 986 CpGs, 829 (84%) were located outside CpG islands which is similar to the total CpGs outside CpG islands from those surveyed (31,316 out of 37,426,14%) suggesting that relatedness does not target CpG islands specifically. Their distribution across the different *Peromyscus* chromosomes is shown in Fig. 7a. Then we calculated if the number of differentially methylated CpGs deviates significantly from their expected number in the corresponding chromosomes if their occurrence was randomly distributed in the different chromosomes. Chromosomes X (*P* = 2.6 × 10^−6^) had significantly fewer while chromosome 4 (*P* = 0.0031) had significantly more than expected CpGs that are differentially methylated according to relatedness (Figs. 7b, 8, and Supplementary Table 4). The most significant deviation was observed in X chromosome.

**Fig. 9.**
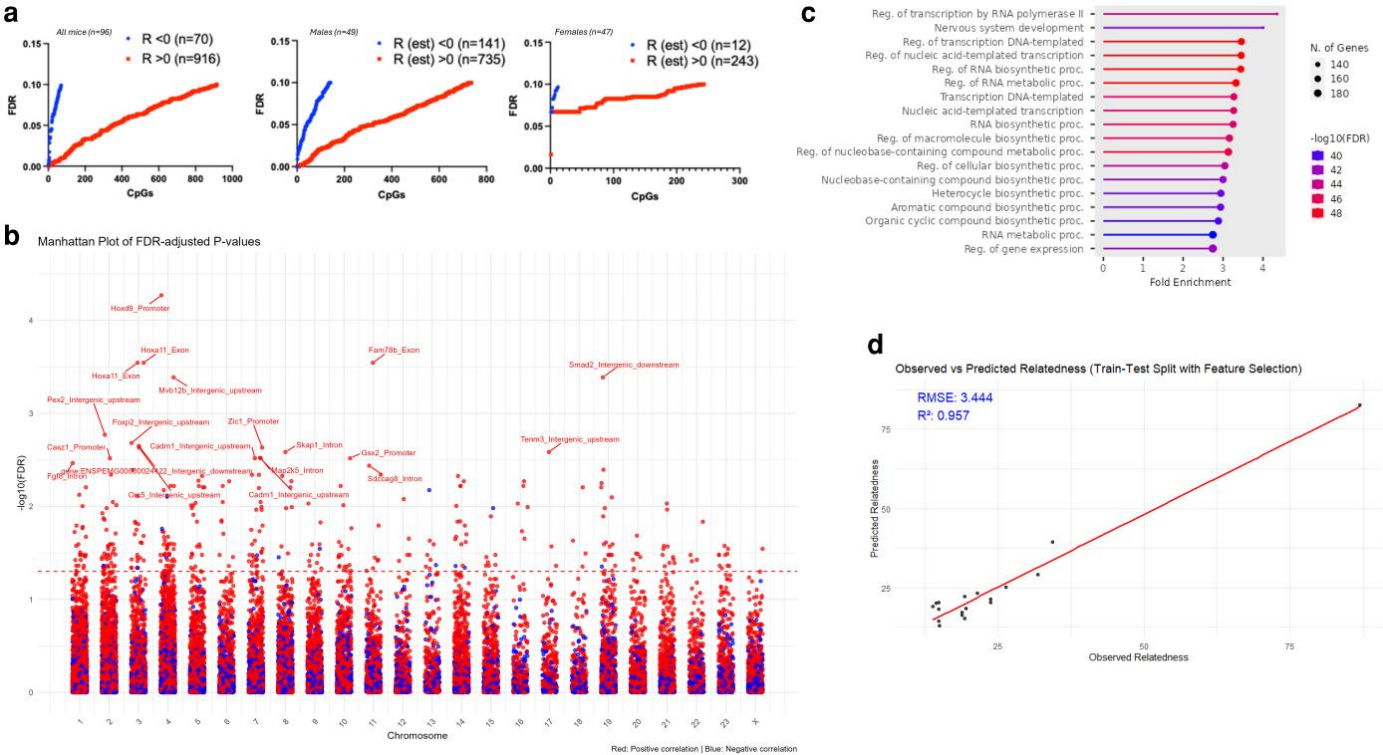
Relatedness-dependent methylation and biological processes. a) Scatter plot showing the number of different CpGs that exhibit positive (R > 0) or negative (R < 0) correlation estimate with relatedness for FDR < 0.1. Scatter plots involving all mice (left), males (middle), or females (right) are shown. b) Manhattan plot of FDR adjusted *P*-values for differentially methylated CpGs at different chromosomes. Red, hypermethylated; Blue, hypomethylated with relatedness. Genes located in the vicinity of the top CpGs are noted. c) GO analysis for biological processes of gene networks affected by relatedness. The analysis includes genes associated with differentially methylated CpGs (FDR < 0.1) related to relatedness. *P* and R values (Pearson's correlation) are shown in the scatter plots. d) A methylation signature for parental relatedness. Observed vs. Predicted Relatedness using Elastic Net Regression with Feature Selection. The plot shows the relationship between observed relatedness values (*x*-axis) and predicted relatedness values (*y*-axis). The red line represents the best-fit regression line. Model performance is evaluated using RMSE = 3.444 and *R*² = 0.957, indicating a strong predictive accuracy.

The vast majority of the relatedness-dependent differentially methylated CpGs were hypermethylated when parental relatedness increased (Fig. 9a). Consistently with the sex-dependent effects in ageing predictions, the effect was 3-fold more pronounced in the males for the hypermethylated and 12-fold more pronounced for the hypomethylated CpGs (Fig. 9a) and contrasts the overall CpG demethylation that occurs with ageing.

Subsequently, we identified the genes that are located in the vicinity of the differentially methylated CpGs and predicted the associated biological processes. Significantly hypermethylated or hypomethylated CpGs and their distribution to different chromosomes is shown in Fig. 9b. By applying *P* < 0.05 and FDR < 0.1, we identified 473 genes that harbored CpGs exhibiting relatedness-dependent methylation. Top gene targets (Supplementary Table 4) were Hoxd9 (*P* = 1.4 × 10^−9^, FDR = 5.3 × 10^−5^) and Hoxa11 (*P* = 2.5 × 10^−8^, FDR = 0.0003), which are involved in neuronal development, neurodegeneration and ageing which supports the proposed link between ageing and parental relatedness (Smith et al. 2018; Turner et al. 2020). Of note is also Pex2 (*P* = 4.1 × 10^−7^, FDR = 0.002) that is involved in peroxisomal function which regulates ROS metabolism (Cipolla and Lodhi 2017). Analysis for biological function pointed to processes such as nervous system development, transcriptional regulation and RNA processing, which is consistent with transcriptional reprogramming (Fig. 9c and Supplementary Fig. 9).

To determine if these chromosomal enrichments were driven by specific genomic loci, we performed a sliding window enrichment analysis (2 Mb windows). We identified distinct sub-chromosomal clusters of relatedness-associated CpGs, most notably a highly significant peak on Chromosome 7 (FDR < 10^−6^) and a secondary peak on Chromosome 10 (FDR < 0.001) (Supplementary Fig. 10, Supplementary Table 5). Annotation of these regions revealed that the Chromosome 7 peak specifically maps to the Zic1/Zic4 locus, while the Chromosome 10 peak encompasses Gsx2. Both loci encode transcription factors critical for neurogenesis and brain development (Corbin et al. 2000; Blank et al. 2011), reinforcing the functional link between parental relatedness and developmental epigenetic programming.

### A methylation signature for relatedness

The aforementioned observations suggest that degree of kinship in the parents can impact CpG methylation by rather consistent manners that can impact biological processes and epigenetic ageing in the offspring. Thus, we asked if a methylation signature can be described that predicts parental relatedness. Out of 369 CpGs with FDR < 0.05, LASSO regression identified 63 CpGs as key predictors (Fig. 9d). The Elastic Net regression model trained on these CpG sites achieved RMSE = 3.444 and *R*² = 0.957, indicating strong predictive performance. A scatter plot of observed vs. predicted values demonstrated high correlation, supporting the reliability of methylation patterns for kinship inference (Fig. 9d).

## Discussion

Genetic and environmental factors determine the individuals' rate of ageing. In the present study, we identify an additional factor that is positioned in the intersection of the two: *Parental relatedness* that has a genetic component because it materializes into the individual's genome, and an “environmental” component because it applies to the genetic identity of the individual's mating partner. The impact of this effect was estimated to be around 13% in the current dataset, in explaining variation in DNA methylation when sex and age were considered and should be further validated by using other datasets. Indeed, our findings persisted when the two outliers of the current dataset were removed, and when an independent dataset was used that involved different *Peromyscus* species.

The effect of parental relatedness may offset negative outcomes of inbreeding and offer advantages to the offspring. Our observations in closed *Peromyscus* stocks that are maintained as outbred genetically diverse colonies for several decades may imply the operation of a mechanism that in part may mitigate potentially negative effects of inbreeding. From that perspective, it is plausible that some harmful consequences of inbreeding may be partially relieved by adaptive changes involving relatedness-dependent shifts in the epigenetic age of inbred offspring and the global CpG hypermethylation to counterweight the overall demethylation that occurs in the genome with ageing. In alignment with the recorded epigenetic age extension due to parental relatedness, it potentially provides a compensatory mechanism according to which hypermethylation due to relatedness offsets the effects of overall demethylation due to ageing (Razin and Riggs 1980; Wilkinson et al. 2022). Thus, if these results do not reflect just an artifact introduced by relatedness that influences epigenetic age estimations, increased relatedness between parents may mitigate inbreeding depression by extending predicted lifespan and by enhancing overall CpG methylation that decreases in ageing. Moreover, our findings demonstrate that the effect of relatedness on epigenetic ageing is not solely direct. Mediation analyses revealed that structured methylome variation, captured by the leading PCs, serves as a pathway through which relatedness exerts its influence on EAA. PC1 and PC2 accounted for ∼25% and ∼32% of the total effect, respectively, with bootstrap and sensitivity analyses supporting robust indirect pathways. Thus, genetic similarity of the parents impacts the methylome in a structured, nonrandom fashion, and that these configurations in turn modulate biological ageing. By contrast, methylome entropy, which also correlates with chronological age, was less influenced by relatedness and likely represents stochastic epigenetic drift. Together, these results suggest a dual model in which heritable forces shape structured epigenetic variation that mediates ageing, while stochastic processes contribute in parallel through entropy-driven disorder.

Sex-stratified mediation analyses revealed sex-dependent effects. In females, PC1 significantly mediated the relationship between relatedness and EAA, whereas in males, PC2 mediated approximately one-quarter of the total effect alongside a direct path of relatedness. This sex dimorphism highlights that males and females may utilize distinct epigenetic architectures to translate genetic similarity into ageing outcomes. Given known sex differences in epigenetic regulation and lifespan (Engelbrecht et al. 2022; Li et al. 2024; Shealy et al. 2025), these findings emphasize the importance of explicitly modeling sex as a biological factor rather than a nuisance covariate.

Chromosome X had fewer while chromosome 4 had more CpGs than expected that exhibited relatedness-dependent methylation. The lower number of relatedness-associated CpGs in X chromosome may be due to the overall hypermethylation of cytosines in X chromosome for X-inactivation. It may also represent an adaptation that offsets the potential presence of X-linked deleterious alleles that cause loss of inbred male offspring (Tarín et al. 2014; Migeon 2020). Indeed, male offspring appear more vulnerable to the harmful consequences of inbreeding depression (Huynh-Dam et al. 2025b).

The role of relatedness in the methylome has implications beyond epigenetic ageing. DNA methylation is a key regulator of gene expression, and our results suggest that parental kinship may act as a modifier of transcriptional profiles. This consideration is particularly relevant when interpreting gene expression or methylation data in genetically diverse populations, where differences in relatedness may confound downstream analyses. Beyond laboratory systems, these insights may be relevant in conservation biology or ancient DNA research. While current kinship inference largely relies on SNPs and short tandem repeats (Kayser and de Knijff 2012; Vai et al. 2020; de Vries et al. 2022), methylation-based inference remains underexplored. Given that methylation signatures are often conserved across species (Haghani et al. 2023; Lu et al. 2023), the development of methylation-based markers of kinship could complement genetic approaches, especially in cases of degraded DNA or incomplete population reference panels.

Several limitations should be considered when these findings are interpreted. For instance, kinship was estimated using pedigree records while heterozygosity calculated by genomic measures such as overall heterozygosity or runs of homozygosity was not considered (Renaud et al. 2019; Shafer and Kardos 2025). Although pedigree-based and DNA-based estimates are generally aligned, their divergence could unveil whether the effects we observed reflected heterozygosity itself or ancestry-related epigenetic imprints. Additionally, our study was conducted in a single tissue (tail) and modest sample size, which limits resolution for sex-stratified analyses. Broader validation across tissues, species, and populations will be essential to determine generalizability. Moreover, the effects of parental relatedness may be confounded by genetic variation patterns passed down to the offspring. In that case, genetic kinship between different mating pairs may result in effects in epigenetic age that are not due to relatedness of the parents *per se* but rather due to specific alleles inherited from their parents. Although this does not seem highly likely because of the lack of clustering of specimens together by a manner that depends on parental relatedness, it remains as a possibility. Finally, it is emphasized that instead of indicating ageing trajectories, relatedness may modulate epigenetic age estimations resulting in overestimation or underestimation, depending on the individuals' parental kinship.

Despite these caveats, our results identify relatedness as a previously unrecognized determinant of epigenetic ageing. In small or structured populations—including laboratory colonies, conservation-relevant species, or human groups with geographic or cultural isolation—relatedness may act as a powerful modifier of epigenetic ageing trajectories. The findings provide proof-of-principle that methylation-based signatures of relatedness exist and can influence biological outcomes. Whether these signatures are species-specific or conserved across evolutionary scales remains an open question. More broadly, our study suggests that relatedness should be considered as a biological variable in studies of epigenetics, ageing, and gene regulation, and may ultimately inform biomedical applications where kinship, methylation, and therapeutic outcomes intersect.

## Supplementary Material

iyaf281_Supplementary_Data

## Data Availability

Raw data for methylation and codes are available in the GitHub repository https://github.com/KimTuyenHuynhDam/Relatedness_pero_DNAm. A permanent archive of the code and datasets used for this publication is available at Zenodo: https://doi.org/10.5281/zenodo.17885513. Methylation data for *P. maniculatus* (BW stock) have been deposited to NCBI Gene Expression Omnibus ID GSE314002 (Horvath and Kiaris 2025). Published methylation data from different *Peromyscus* species were retrieved from GSE223748 (Horvath et al. 2022). Supplemental material available at GENETICS online.
