# Supplementary material for: Parental kinship influences global methylation and epigenetic age estimation in Peromyscus
Source: Genetics. 2025 Dec 29;232(3):iyaf281. doi: 10.1093/genetics/iyaf281 (PMC13017600; doi:10.1093/genetics/iyaf281)
Supplement: iyaf281_Supplementary_Data [file iyaf281_supplementary_data.zip › Supplementary_Figures_GENETICS-2025-308888.pdf]

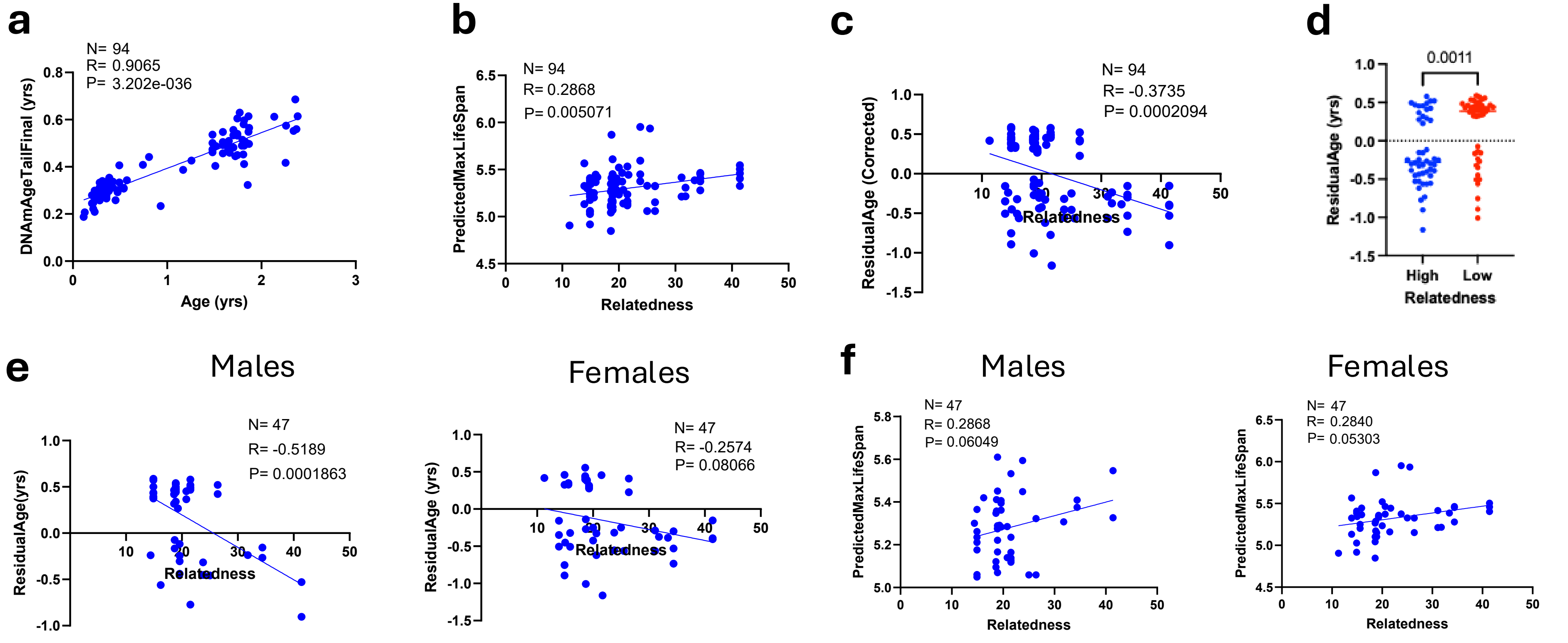

Supplementary Figure 1. Methylation clock for *P. maniculatus* and predicted lifespan with 2 outliers removed. Data are the same with those described in Figure 1 but the 2 outliers with very high relatedness were omitted from the analysis. Therefore n=94. **a.** Validation of the epigenetic clock for *P. maniculatus* (BW stock). The X axis shows the animals' biological age while the Y axis shows the predicted epigenetic age (DNAmAge) of the animals. **b.** Scatter plot showing the correlation between predicted lifespan (Y axis) and relatedness (X axis). **c.** Correlation between age residual and relatedness in *P. maniculatus*. **d.** Residual age in animals having equal or above (high) (n=48) or below (low) (n=46) median relatedness are shown. P-value (student's t-test)' is indicated. **e.** Correlation between age residual (Y axis) and parental relatedness (X axis) in male or female P. maniculatus. **f.** Scatter plot showing the correlation between predicted lifespan (Y axis) and relatedness (X axis) in males (left) and females (right) P. maniculatus. P and R values (Pearson's correlation) are shown in the scatter plots. Epigenetic age and calculations of predicted lifespan were adjusted for average chronological age (see Methods for more details).

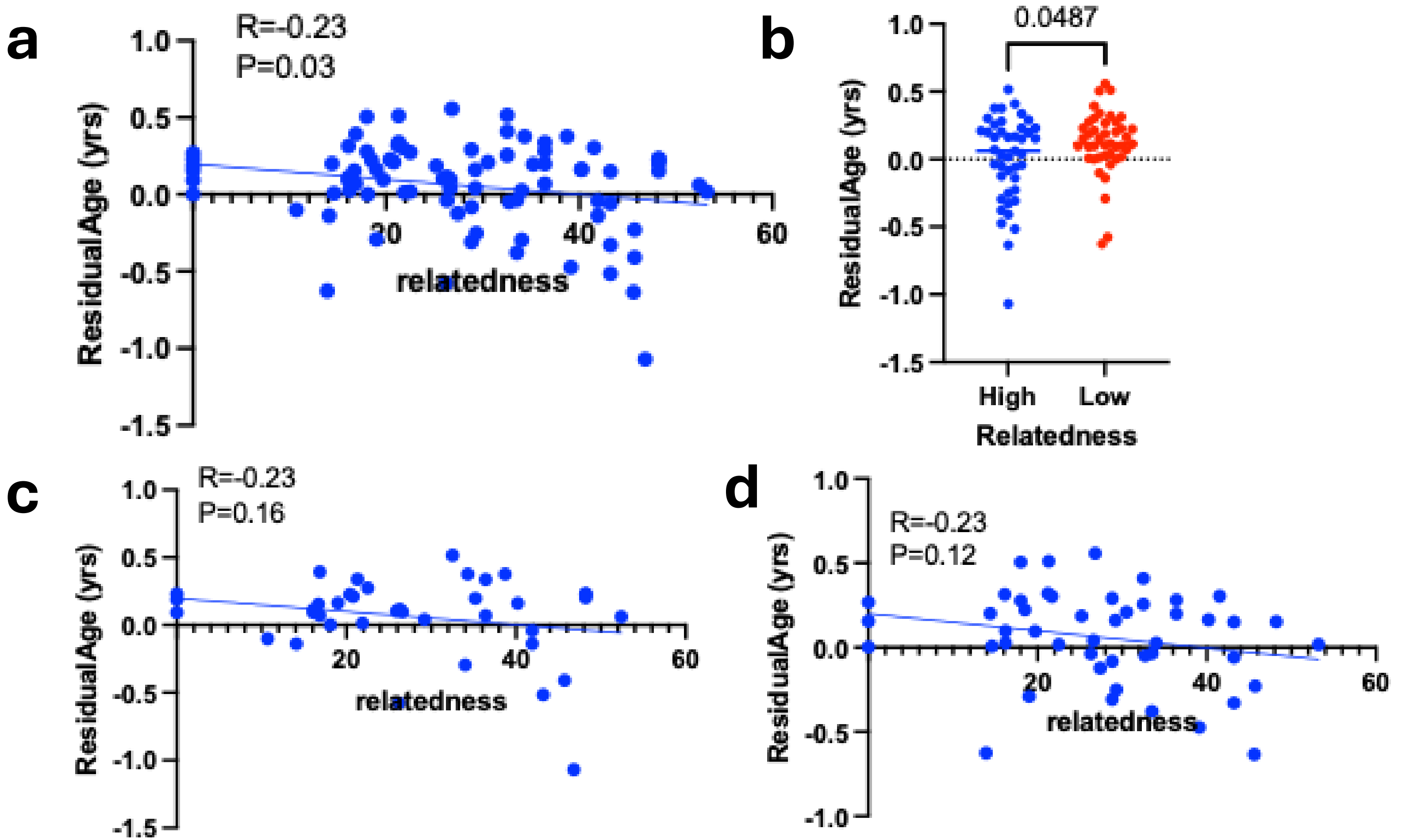

Supplementary Figure 2. Residual age and relatedness in 6 Peromyscus stocks. **a.** Correlation between age residual (Y axis) and parental relatedness (X axis) in Peromyscus (6 stocks and 6 F1 POXBW hybrids as described earlier (Horvath et al, 2021). **b**. Residual age in animals above (high) (n=43) or below (low) (n=43) median relatedness. P, (student's t test) is indicated. Age residual plotted against parental relatedness for males **(c)** and females **(d)**. P and R values (Pearson's correlation) are shown in the scatter plots.

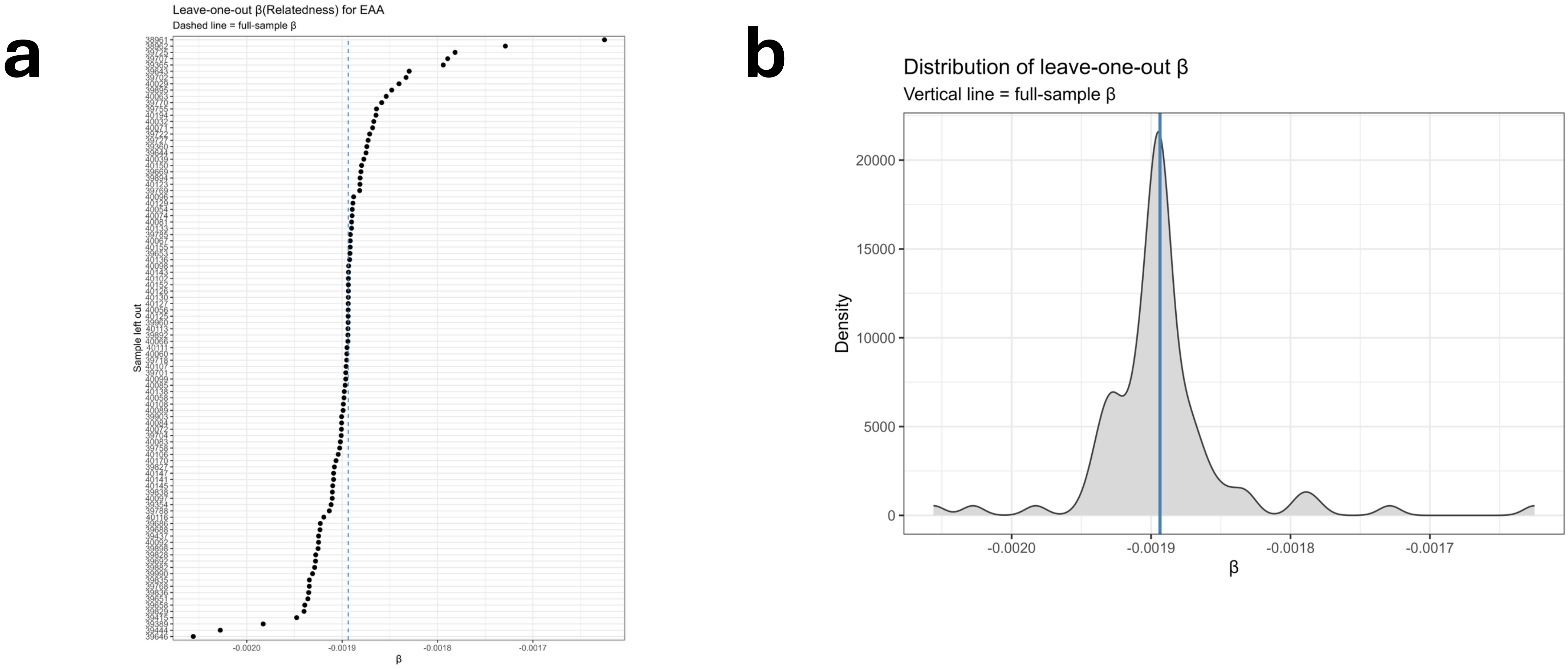

Supplementary Figure S3. Leave-one-out influence analysis for the relatedness–EAA association

a. Leave-one-out estimates of the regression coefficient for relatedness from the model *EAA ~ relatedness + age + sex*. Each point is the β obtained after refitting the model with one individual removed (y-axis labels the omitted individual). The dashed vertical line marks the full-sample β.

b. Kernel density of the leave-one-out β values; the vertical line marks the full-sample β. Across all refits, the association remains negative (median β ≈ −0.00189; IQR [−0.00206, −0.00162]), indicating that no single individual drives the result and supporting a robust negative association between relatedness and epigenetic age acceleration.

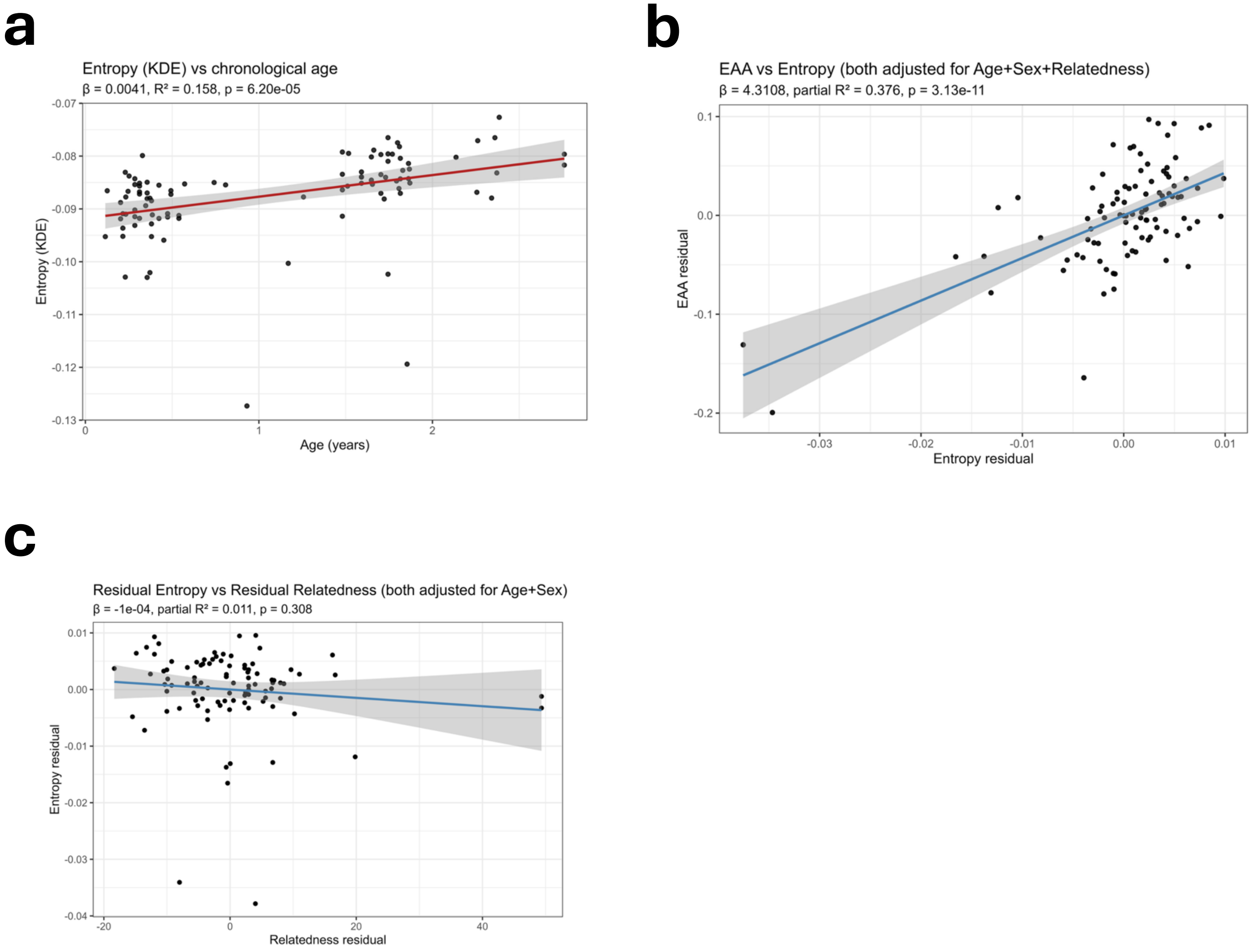

Supplementary Figure S4. Epigenetic entropy reflects age-related methylome disorder.

a. Entropy (global methylome disorganization) increases with chronological age (linear regression with 95% CI shading).

b. EAA is positively associated with entropy when both are adjusted for age, sex, and relatedness (partial regression; line with 95% CI).

c. No evidence that entropy relates to genetic relatedness after adjusting for age and sex (residual-on-residual plot with 95% CI).

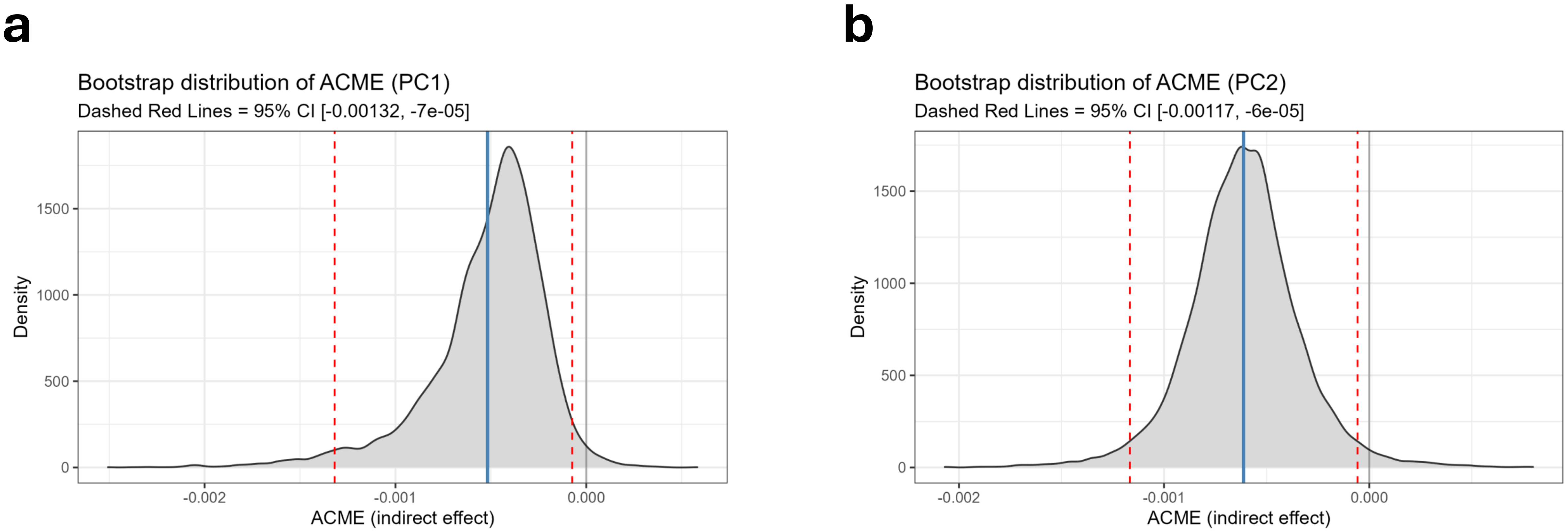

Supplementary Figure S5. Bootstrap distributions of indirect effects (ACME) for methylome PCs

a. **PC1 mediator**. Nonparametric bootstrap distribution (n=10,000) of the average causal mediation effect (ACME). The solid blue vertical line marks the point estimate; dashed red lines mark the exact 95% bias-corrected bootstrap Confidence Interval (CI). The upper bound of the CI is strictly less than zero (-7e-05), confirming a significant negative indirect effect.

b. **PC2 mediator.** As in (a) for PC2. The 95% CI upper bound is -6e-05, excluding zero and supporting a robust negative mediated pathway via PC2.

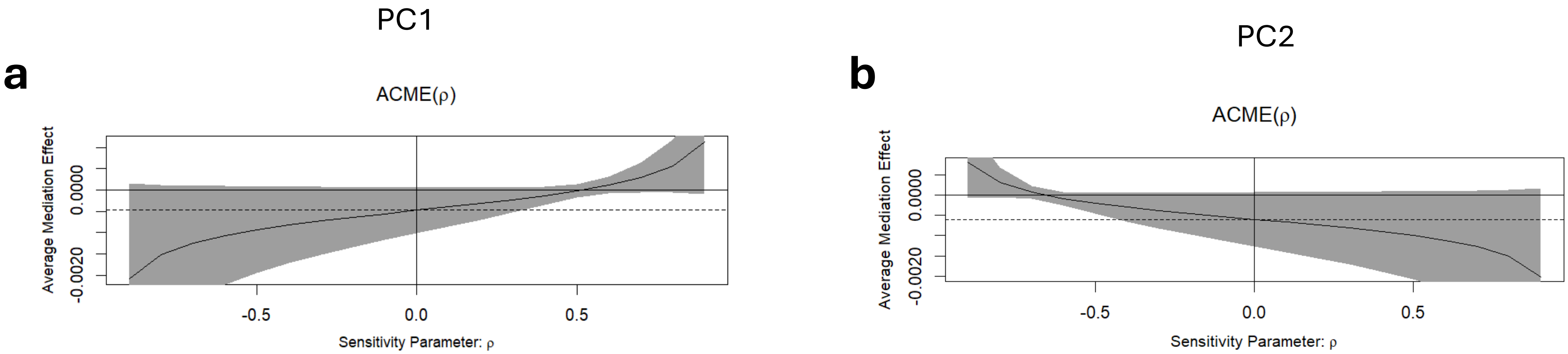

Supplementary Figure S6. Sensitivity of the mediated effect (ACME) to unmeasured confounding

a. **PC1 mediator.** Imai–Keele–Tingley sensitivity analysis plotting ACME against the assumed correlation (ρ) between mediator and outcome error terms. The shaded band is the 95% CI. The upward slope indicates that the ACME is most sensitive to positive unmeasured confounding. However, across a broad range of ρ (approx. -0.9 to 0.5), the ACME remains significantly negative, indicating that moderate levels of unmeasured confounding would not overturn the inference.

b. **PC2 mediator.** As in (a) for PC2. The downward slope reflects sensitivity to negative unmeasured confounding**.** Despite this contrasting pattern, the ACME curve remains significantly negative for a wide interval of ρ (approx. -0.4 to 0.9), further supporting the robustness of the mediated pathway via PC2.

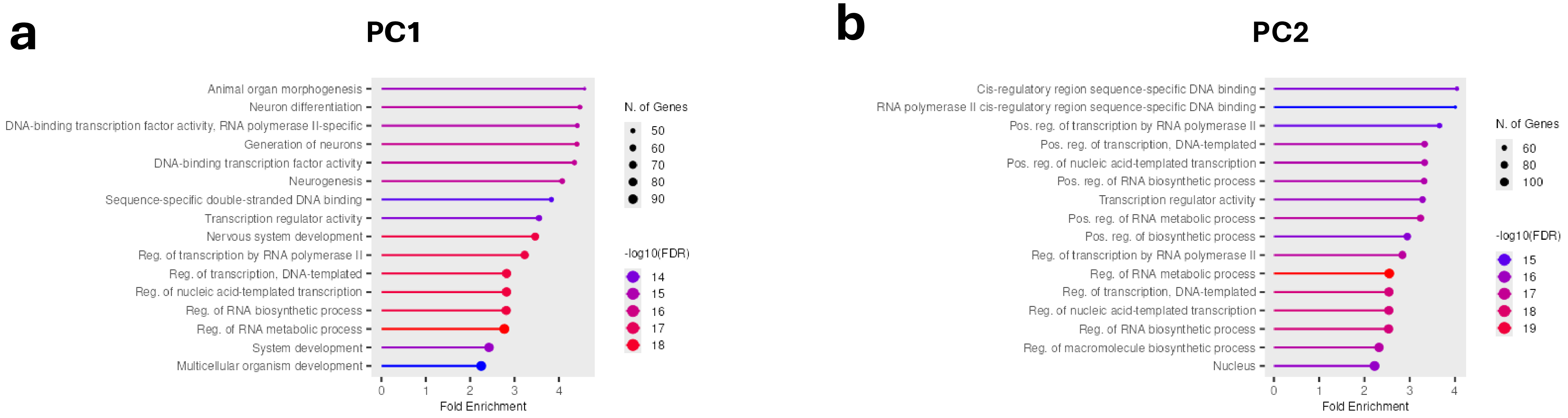

Supplementary Figure S7. Gene Ontology enrichment of top CpGs contributing to PC1 and PC2. CpGs with the highest absolute loadings from (A) PC1 and (B) PC2 (top 369 each, to match the number of significant relatedness-associated CpGs) were mapped to nearby genes and analyzed for functional enrichment. PC1 CpGs were enriched for developmental processes, including *animal organ morphogenesis, neuron differentiation, and nervous system development*, as well as DNA-binding transcription factor activity. In contrast, PC2 CpGs were enriched for *cis-regulatory region binding, RNA polymerase II-specific transcriptional regulation, and RNA biosynthetic processes*. Node size reflects the number of genes in each category, while color indicates enrichment significance (–log10 FDR). These results suggest that PC1 captures a broad developmental methylome program, whereas PC2 highlights regulatory CpGs involved in transcriptional control.

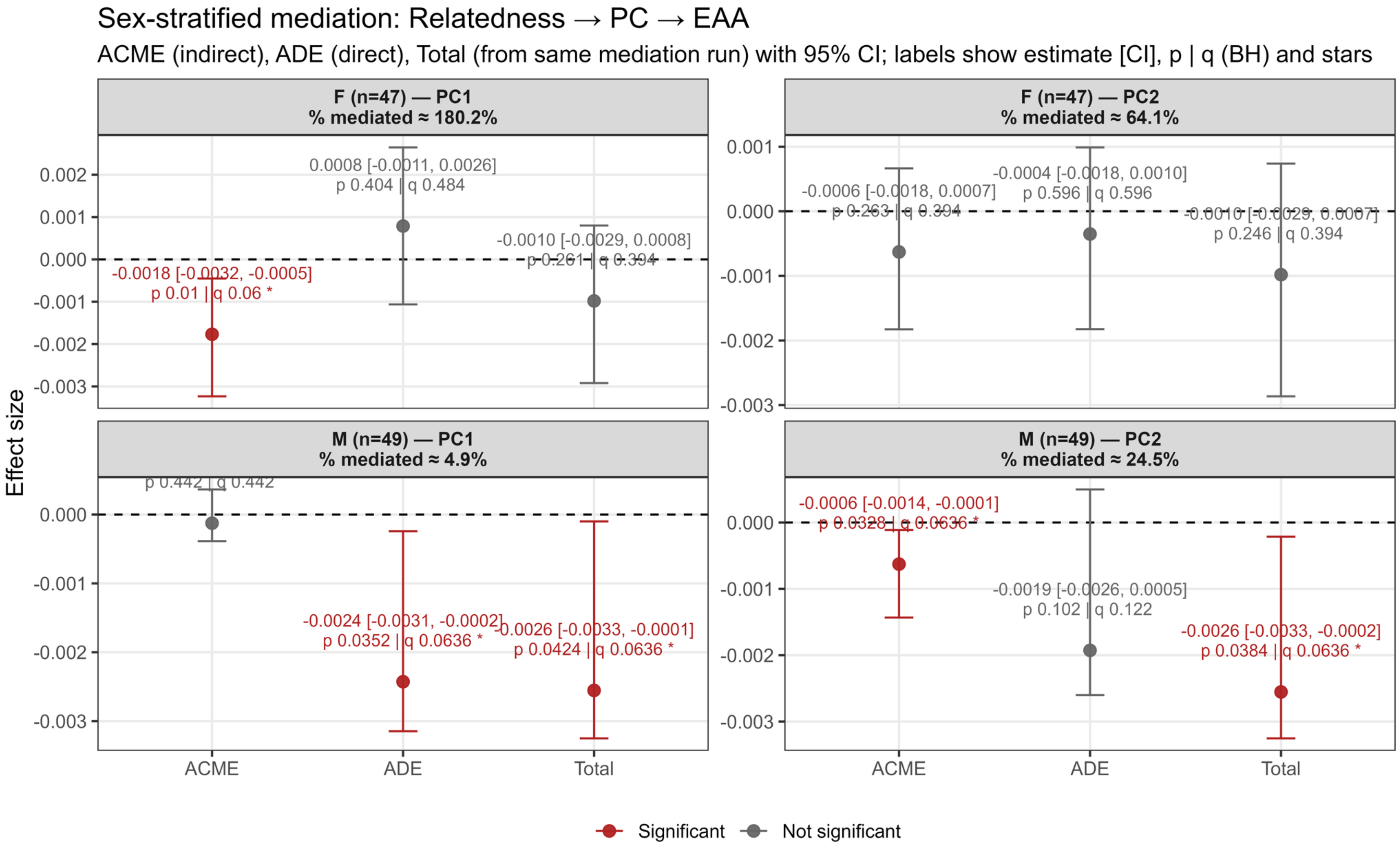

Supplementary Figure S8. Sex-stratified mediation of relatedness → EAA via PC1/PC2. Points = ACME (indirect), ADE (direct), Total (all from the same mediation run); bars = 95% CI. Labels show estimate [CI], raw p | BH q (by sex) and significance (*** <0.001, ** <0.01, * <0.05).

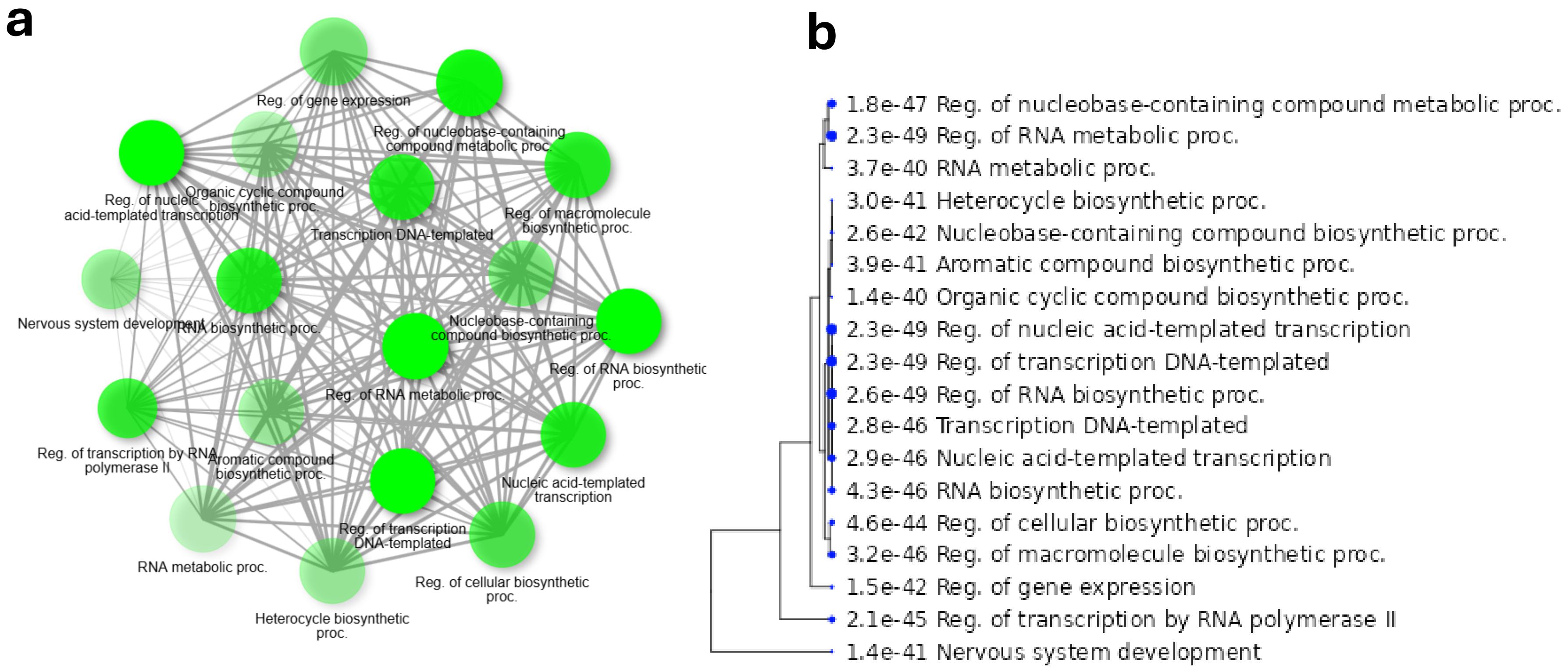

Supplementary Figure 9. Differentially methylated genes and relatedness. a. Gene networks impacted by parental relatedness (FDR < 0.01). **b**. Hierarchical clustering tree of significant pathways.

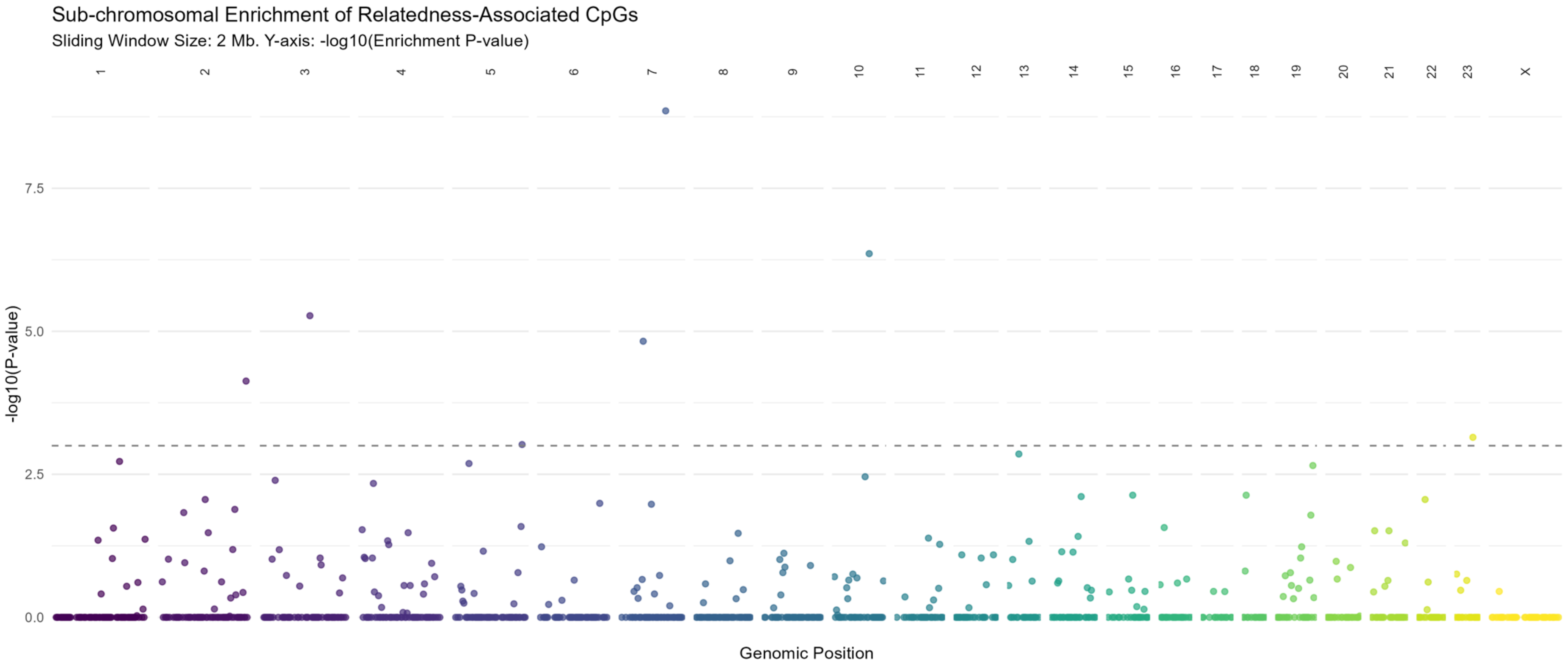

Supplementary Figure 10. Sub-chromosomal enrichment of relatedness-associated CpGs. A sliding window analysis (2 Mb window size) was performed to identify genomic regions significantly enriched for relatedness-associated CpGs. The Manhattan-style plot displays genomic position (x-axis) versus enrichment significance (-log10(P-value), y-axis). Significant clusters are observed on several chromosomes, with the most prominent peaks located on Chromosome 7 (mapping to the *Zic1*/*Zic4* neurodevelopmental locus) and Chromosome 10 (mapping to the *Gsx2* homeobox gene). The dashed line represents the significance threshold.
