# Supplementary material for: Parental kinship influences global methylation and epigenetic age estimation in Peromyscus
Source: Genetics. 2025 Dec 29;232(3):iyaf281. doi: 10.1093/genetics/iyaf281 (PMC13017600; doi:10.1093/genetics/iyaf281)
Supplement: iyaf281_Supplementary_Data [file iyaf281_supplementary_data.zip › Supplementary_Tables_Legends_GENETICS-2025-308888.docx]

**Legends of Supplementary Tables**

**Supplementary Table S1.** Leave-one-out regression results for relatedness predicting EAA. Per-individual leave-one-out refits of EAA ~ relatedness + age + sex. For each omitted individual, the table reports the estimated β for relatedness (leave-one-out), its standard error, 95% CI, and p-value, along with the full-sample β for reference.

**Supplementary Table S2.** Top CpGs contributing to PC1 and PC2 and their overlap with relatedness-associated CpGs. CpGs were ranked by absolute loadings from the PCA rotation matrix, and the top 369 CpGs were selected for each component to match the number of significant relatedness-associated CpGs. Sheet “PC1”: List of the top 369 CpGs contributing to PC1, with genomic annotation. Sheet “PC2”: List of the top 369 CpGs contributing to PC2, with genomic annotation. Sheet “overlapped CpGs”: Summary of the number of overlapping CpGs between PC1 or PC2 and the relatedness-associated CpG set (PC1 ∩ Relatedness = 0; PC2 ∩ Relatedness = 9).

**Supplementary Table S3.** Sex-specific causal mediation results for the pathway Relatedness → (PC1 or PC2) → EAA. For each Sex × Mediator cell, the table lists ACME (indirect), ADE (direct), and Total effects with 95% bootstrap CIs (10,000 draws), raw p, BH-adjusted q (within sex), significance stars (*** <0.001, ** <0.01, * <0.05), and n. “% mediated” is computed from the same model as ACME / Total × 100; values may exceed 100% or be undefined when the total effect is small or signs differ (inconsistent mediation).

**Supplementary Table S4**. List of CpGs that exhibit methylation correlated with relatedness in *P. maniculatus* in all animals (sheet 1), males (sheet 2), females (sheet 3). Gene list associated with the CpGs of sheet 1 and have FDR<0.1 are shown in sheet 4. Distribution of CpGs methylated differentially according to relatedness are shown in sheet 5.

**Supplementary Table S5. Significant sub-chromosomal clusters of relatedness-associated CpGs and mapped genes.** Results from a sliding window enrichment analysis (2 Mb windows) identifying genomic loci significantly enriched for relatedness-associated CpGs.
